# Price tag of glaucoma care is minor compared with the total direct and indirect costs of glaucoma: Results from nationwide survey and register data

**DOI:** 10.1371/journal.pone.0295523

**Published:** 2023-12-20

**Authors:** Petri K. M. Purola, Joonas Taipale, Saku Väätäinen, Mika Harju, Seppo V. P. Koskinen, Hannu M. T. Uusitalo

**Affiliations:** 1 Department of Ophthalmology, Faculty of Medicine and Health Technology, Tampere University, Tampere, Finland; 2 Finnish Register of Visual Impairment, Finnish Federation of the Visually Impaired, Helsinki, Finland; 3 ESiOR Oy, Kuopio, Finland; 4 Department of Ophthalmology, University of Helsinki and Helsinki University Hospital, Helsinki, Finland; 5 Department of Public Health and Welfare, Finnish Institute for Health and Welfare, Helsinki, Finland; 6 Tays Eye Center, Tampere University Hospital, Tampere, Finland; Seirei Hamamatsu General Hospital, JAPAN

## Abstract

**Background:**

The estimations of the economic burden of glaucoma have focused on comparing different treatment modalities; hence, the total direct and indirect costs of glaucoma at population level are not well known.

**Objective:**

To estimate the direct and indirect costs of glaucoma and its treatment in Finland.

**Methods:**

Economic and glaucoma data were collected from the cross-sectional nationwide Health 2000 health examination survey linked to multiple national registers, which allowed a 13-year follow-up between 1999–2011 among survey participants. Direct costs covered eye- and non-eye-related hospitalizations and outpatient visits, outpatient health care services, and travel costs among participants aged 30 years or older, adjusted for age and sex. Indirect costs covered premature retirement and productivity losses among participants aged 30–64 years. Glaucoma patients (*n* = 192) were compared with non-glaucomatous population (*n* = 6,952).

**Results:**

The annual additional total direct costs were EUR 2,660/glaucoma patient, EUR 1,769/glaucoma patient with medication, and EUR 3,979/operated glaucoma patient compared with persons without glaucoma. The respective additional total indirect costs were EUR 4,288, EUR 3,246, and EUR 12,902 per year. In total, the additional annual direct and indirect expenditures associated with glaucoma in Finland were EUR 202 million (0.86% of total expenditures of health care) and EUR 71 million (0.03% of the Finnish gross domestic product) arising mainly from non-eye-related hospitalizations and productivity losses, respectively.

**Conclusion:**

Glaucoma is associated with an increased health care consumption mainly due to non-eye-related health care, which can be explained by the vision loss as well as increased number of co-morbidities among glaucoma patients. Therefore, glaucoma constitutes a major economic burden for the health care system and society, highlighting the importance of early glaucoma interventions. The difference in direct and indirect costs between glaucoma treatment groups is explained by the uneven distribution of co-morbidities.

## Introduction

Glaucoma is an optic neuropathy characterized by progressive degeneration of retinal ganglion cells. Globally, over 70 million individuals suffer from glaucoma [1]. In Finland, there are over 80,000 glaucoma patients, of which approximately 8% are visually impaired with visual acuity (VA) lower than 0.3 (Snellen decimals) [2, 3]. The prevalence of glaucoma is increasing globally due to the rapidly growing number of older people [1, 2, 4]. Other risk factors for glaucoma besides age include elevated intraocular pressure, family history, presence of exfoliative material, myopia, and African ethnicity [5]. Currently, there are three types of glaucoma treatments: drugs, surgical procedures, and laser treatments [6]. Even though timely and effective treatment could prevent the deterioration of vision, glaucoma remains as one of the leading causes of blindness worldwide. Furthermore, the low public awareness, the asymptomatic early stages of glaucoma, and the non-adherence to prescribed therapy can lead to inadequate control of glaucoma, with severe consequences for both the individual and the society [7].

Given the social consequences of glaucoma and the limited resources available to health care providers, it is crucial to provide appropriate information to facilitate the decision making and the allocation of health care resources. However, the impact of glaucoma on total direct and indirect costs at population level is not well known. Majority of the previous glaucoma-related publications have focused on comparing different treatment modalities at clinical settings [8–11]. Few studies have estimated either direct or indirect costs of glaucoma and its treatment [12–15].

Hence, there is a need for a comprehensive picture of the economic burden of glaucoma including all eye- and non-eye-related direct and indirect costs associated with the disease—for example, hospitalizations due to falls and injuries. More population-wide studies are also required to corroborate the previous findings and to provide accurate estimates of the costs in different nationwide settings. Furthermore, the use of multiple data sources, such as national surveys and registers, is uncommon, even though it could provide more accurate estimates on the use of health care services and both direct and indirect costs. Therefore, our aim was to evaluate the economic impact of glaucoma and its treatment on the Finnish society by combining the data of a nationwide health examination survey and national health registers, estimating both direct and indirect costs associated with the disease.

## Materials and methods

### Study design, data, and population

The Finnish Institute for Health and Welfare (THL) conducted the nationwide Health 2000 survey which collected comprehensive information on health and well-being in Finland during 2000–2001 [16]. The representative sample of the Finnish adult population was selected by utilizing a probability-clustered sampling and weighting scheme. The survey included a face-to-face interview, self-administered questionnaires, and a thorough health examination. The sample included 8,028 subjects aged 30 years and older, and the unweighted participation rate was 93%. The sample weights were calibrated by post-stratification, defined by age, sex, region, and native language to account for non-response and missing data. The details of the survey methods have been published elsewhere [16].

Information on the use of outpatient health care services was collected in the interview, including the number of private, occupational, health center, and other doctor visits, and the number of occupational, home care, and outpatient nurse visits during the preceding 12 months.

The habitual distance VA was measured in the health examination by an educated study nurse binocularly at 4 m. Illumination was set to ≥ 350 lx on the modified logMAR letter chart. All VA values are presented as Snellen decimals. Low VA values outside the modified logMAR letter chart that could not be determined were reported as 0.01. Based on previous studies [17, 18], distance VA was classified into following groups: VA ≥ 1.0 (good vision), VA 0.63–0.8 (adequate vision), VA 0.32–0.5 (weak vision), and VA ≤ 0.25 (visual impairment).

The survey sample was linked to national registers. Data on entitlements to reimbursement for glaucoma medication (during 1965–2011) and the number of glaucoma medication prescriptions (ATC S01E; 1999–2011) of the survey participants were obtained from the registers maintained by the Social Insurance Institution of Finland (Kela). Data on the diagnoses and operations of the survey participants were obtained from the Care Registers for Social Welfare and Health Care maintained by the Finnish Institute for Health and Welfare. The care register data covered inpatient care visits (Hilmo, 1968–2011), which included the number and length of hospitalizations, and specialized health care outpatient visits (AvoHilmo, 1997–2011). A follow-up time was calculated for each participant separately to account for the survival of the participants. The period of scrutinization was extended to 13 years (1.1.1999–31.12.2011) to represent the mean annual usage more accurately. The follow-up durations were corrected for participants who had died during the follow-up period (*n* = 1,279) with a range of 1.2–13.0 years. We included all eye- and non-eye-related hospitalizations and outpatient visits. Eye-related hospitalizations and visits were considered those with main diagnosis H00–H59 International Classification of Diseases (ICD) version 10.

Information on the status and time of retirement were collected in the interview of the Health 2000 survey. To improve the quality of the retirement data and to account the follow-up, additional retirement information were acquired from the Health 2011 health examination survey [19], a follow-up to the Health 2000 survey conducted in Finland in 2011–2012, for participants who partook at both time points.

Based on the Hilmo/AvoHilmo and Kela register data, survey participants were classified into three glaucoma groups following the same procedure as in our previous study [2]: glaucoma, all; glaucoma treated with medication; and operated glaucoma. Laser treatments were not included as a separate group. Survey participants who did not belong to these groups were considered to not have glaucoma and were classified as glaucoma negatives. The details of the classification are shown in Table 1. We analyzed participants who had either survey visits available or both survey and Hilmo/AvoHilmo visits available.

**Table 1:** Classification of glaucoma.

**Glaucoma, all**	Entitlement to special reimbursement for glaucoma medication between 1965–2000 (Kela data) OR High number (> 10) of glaucoma medication prescriptions between 1999–2000 (Kela data) OR Glaucoma diagnosis^*a*^ between 1968–2000 (Hilmo/AvoHilmo data) OR Eye operation^b^ due to glaucoma between 1997–2000 (Hilmo/AvoHilmo data)
**Glaucoma, medication**	Glaucoma and glaucoma medication prescriptions between 1999–2000 (Kela data)
**Glaucoma, operated**	Glaucoma and eye operation^b^ due to glaucoma between 1997–2000 (Hilmo/AvoHilmo data) OR Glaucoma and self-reported glaucoma operation in the Health 2000 survey interview
**Glaucoma negatives**	No glaucoma based on the register data before 31.12.2011 or death AND No self-reported glaucoma based on the Health 2000 Survey interview

Hilmo/AvoHilmo = inpatient/outpatient visits in the Care Registers for Social Welfare and Health Care, Kela = Social Insurance Institution of Finland

^a^International Classification of Diseases diagnosis codes 37500–37520, 37598–37599 for version 8, 3651–3659 for version 9, and H40, H40.1–H40.9 for version 10

^b^At least one of the following: trabeculectomy and iridectomy, glaucoma shunt operation, nonpenetrating glaucoma surgery, other filtering operation, and transscleral laser coagulation of ciliary body

### Cost analysis

This economic evaluation was performed in accordance with the CHEERS 2022 guidelines (S1 Appendix) [20]. We utilized a prevalence-based bottom-up approach to assess both the direct and indirect costs associated with glaucoma. The direct costs were based on registered hospitalizations and outpatient visits, self-reported outpatient health care services, and travel costs for outpatient visits during the follow-up. Unit costs were converted to 2019 level in the analyses based on the most recent estimates on health expenditure and financing in Finland [21–23], and they are listed in S1 Table. Public health care costs included laboratory, administrative, and other collateral costs. For private practitioners, we examined mean administrative costs of three major private health care service providers and in the analyses, we applied weighted average according to the market shares. The proportions of emergency and non-emergency visits have been applied to the unit costs for outpatient visits based on Sotkanet-database and the outpatient visit data from Pirkanmaa Hospital District: in 2019, the proportions of emergency visits were 37.2% in primary health care, 9.8% in specialized health care, and 8.4% specifically for ophthalmologists. Based on the features of Finnish health care system, current proportions are the most precise estimates we can provide. The unit costs do not include the customer fees as our focus was on societal costs. Drug costs and direct non-health care costs excluding transportation were not included in the study as appropriate data were not available. The calculation of travel costs for outpatient visits has been described previously [22].

The indirect costs comprised premature retirement and related productivity losses. The number of premature retirement years was calculated for each person with known time of retirement, starting from age of 30 years up to 64 years. If the person was known to have retired, but the time of retirement was not known, the average retirement age in the population was used instead, separately for glaucoma groups (59.5 years, *n* = 11) and glaucoma negatives (57.4 years, *n* = 268). If the person was older at the time of the survey than the average age, the age at the time of the survey was used (Health 2000 or 2011, 42 glaucoma negatives). If the person had died before age of 65 years during the follow-up, the years were calculated up to age at death. If the person was younger than 65 years during the follow-up, the years were calculated up to age in 2011. Productivity losses were calculated using the premature retirement years. The annual indirect costs were estimated by dividing the total costs by the mean duration of working career in Finland (32.6 years in 2011) [24]. The indirect costs were also converted to 2019 euros in the analyses (S1 Table). Because this is a retrospective population-based study, intangible costs such as pain and suffering and care provided by nonpaid caregivers were not included in the analyses.

### Statistical methods

All data were analyzed with R software (v. 4.2.1, R Core Team, R Foundation for Statistical Computing, Austria). The sampling design of the survey was accounted for using Survey package 3.37 for R [25] and weighting scheme calculated by the Finnish Institute for Health and Welfare. One glaucoma negative and one verified glaucoma patient were excluded from further analyses as high outliers. Age- and sex-adjusted costs as well as non-adjusted costs were calculated. We estimated the total costs at population level by applying the weights. As the data were continuous and quantitative, we calculated means, standard deviations, and standard errors. Because the distribution of the data was right-skewed, we used the Kruskal–Wallis test for multiple comparisons, adjusted with the Dunn–Bonferroni correction from package DescTools 0.99.44 [26]. Pearson correlation coefficients were calculated using jtools package 2.1.4 [27], which is an increment to the Survey package that accounts for the sampling design. For all analyses, a two-tailed p value of < 0.05 was considered as statistically significant.

To account for different co-morbidities and other confounders, we applied generalized linear models to evaluate the total direct and indirect costs. The self-reported co-morbidities were collected from the Health 2000 survey interview data, and they included unoperated cataract, retinal degeneration, heart diseases, pulmonary diseases, vascular diseases, musculoskeletal conditions, hypertension, diabetes, psychiatric disorders, Parkinson’s disease, and unspecified cancer. The co-morbidities were selected and grouped according to our previous publications [17, 18]. Other confounders were age, sex, and visual impairment (distance VA ≤ 0.25). Because the cost data were right-skewed and the proportion of participants with zero costs was under 20% [28], we applied Tweedie distribution using gamma with log link scale response which showed the best fit using package statmod 1.4.36 [29]. We used both forward and backward stepwise methods to evaluate the fitness of the generalized linear model, and for the final analysis we chose a model with non-eye-related co-morbidities. We estimated the marginal means and contrasts using package emmeans 1.7.3 [30].

### Ethics approval and informed consent

The Health 2000 Survey was approved by the Coordinating Ethics Committee at the Hospital District of Helsinki and Uusimaa in Finland [16]. The survey was conducted in accordance with the ethical standards of the institutional and national research committees, and with the 1964 Helsinki declaration and its later amendments or comparable ethical standards. Written informed consent was obtained from all participants [16].

## Results

Of the 8,028 members of the Health 2000 survey sample, 7,367 (91.8%) had information available on glaucoma status and both direct and indirect costs. Of the 192 study participants who had economic data available and had glaucoma, 141 were treated with medication, 59 were treated with surgery (of which 39 were also treated with medication), and 31 had no known treatment. Details of the study population are summarized in Table 2.

**Table 2:** Summary of the health 2000 study population aged 30 year and older.

	*n*	% women	Mean age (years; SD)
Eligible sample	8,028	55	54 (16)
Direct and indirect costs known	7,368^a^	55	54 (16)
Glaucoma status known	7,367	55	54 (16)
Glaucoma, all	192	71	74 (11)
Glaucoma, medication	141	73	74 (11)
Glaucoma, operated	59	68	75 (12)
Glaucoma negatives	6,952	54	53 (16)

SD = standard deviation

^a^Four persons had missing data on retirement status

**Fig 1:**
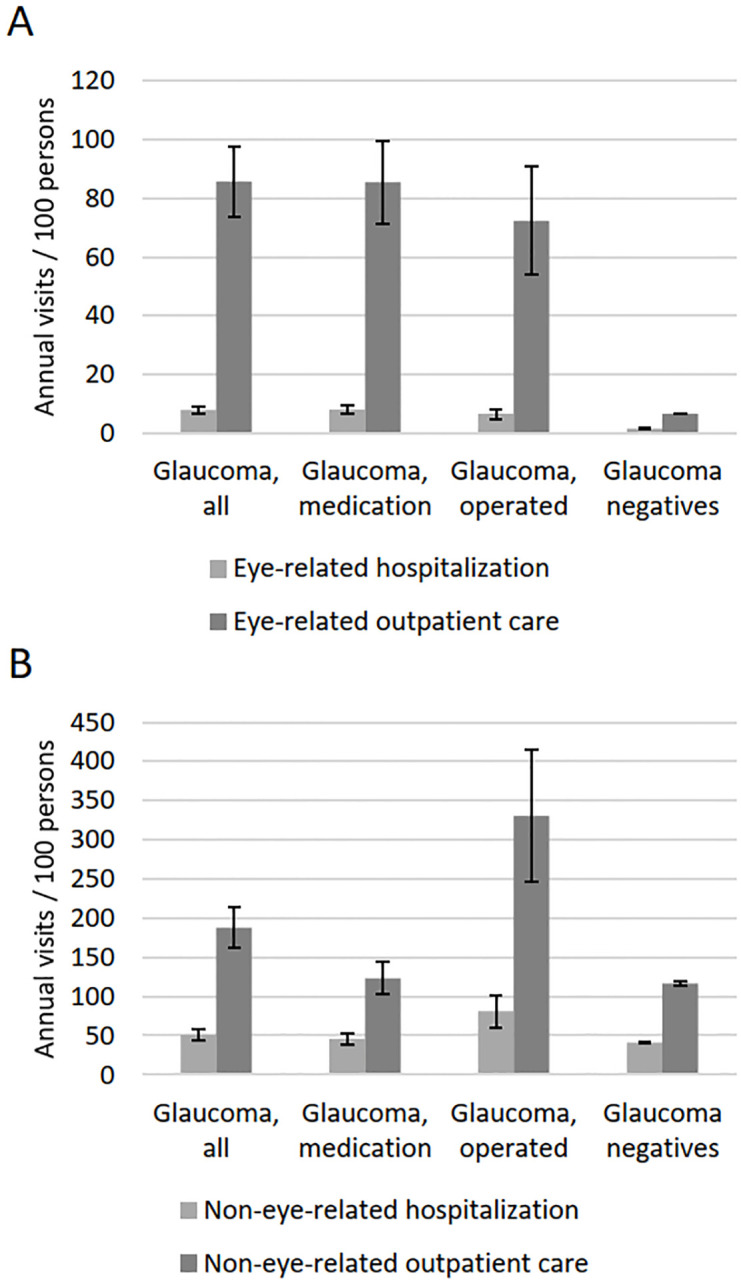
Average eye-related (A) and non-eye-related (B) hospitalizations and outpatient visits per year adjusted for age and sex with 95% confidence intervals. Differences between glaucoma groups and glaucoma negatives were statistically significant (p < 0.001). There were no significant differences within glaucoma groups. Data on hospitalizations and outpatient visits were collected during 1999–2011.

**Fig 2:**
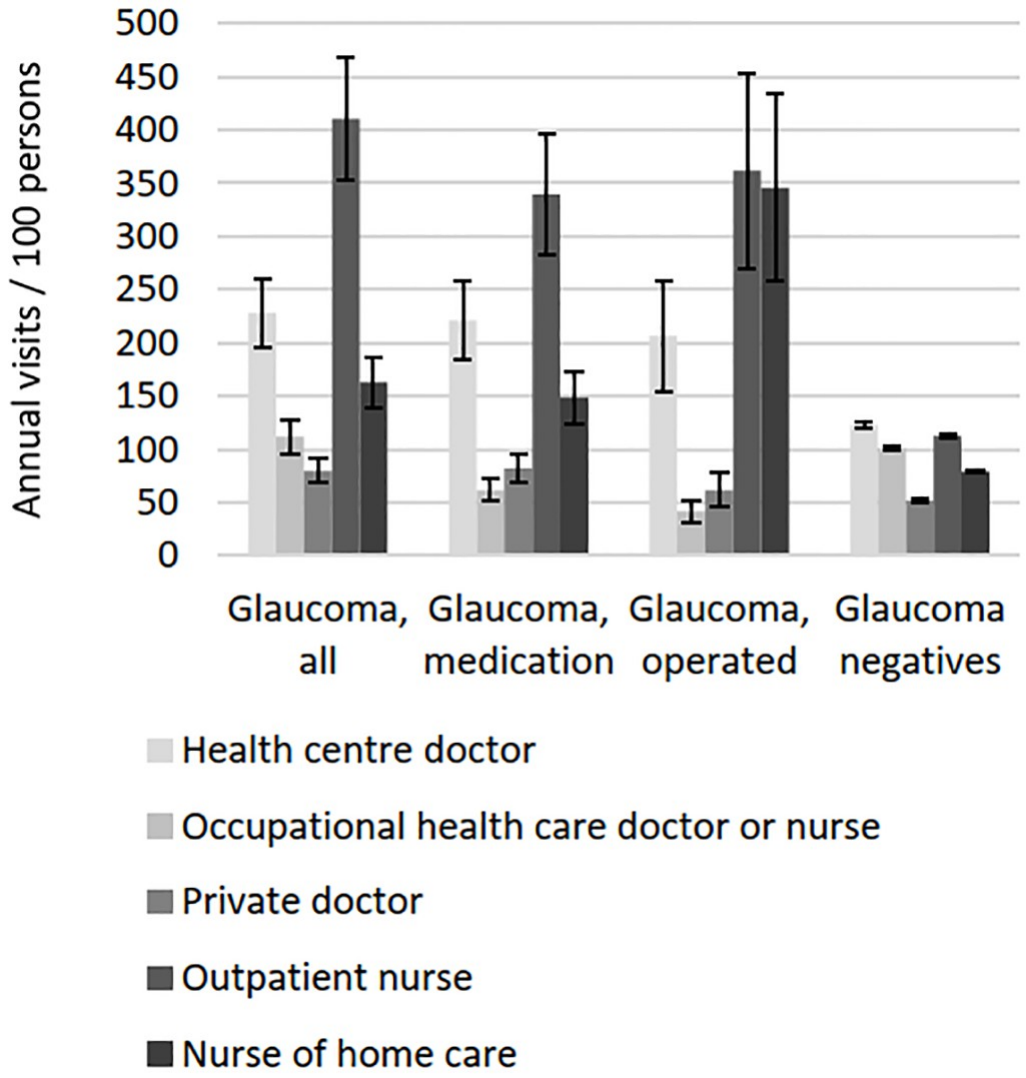
Average self-reported use of outpatient health care services in the year 2000 adjusted for age and sex with 95% confidence intervals. Differences between glaucoma groups and glaucoma negatives were statistically significant (p < 0.001). There were no significant differences within glaucoma groups.

All glaucoma groups showed significantly higher number of both eye-related and non-eye-related hospitalizations and outpatient visits than persons without glaucoma even after adjusting for age and sex (p < 0.001; Fig 1). Outpatient care was more frequent than inpatient care among both glaucomatous and non-glaucomatous subjects. Five percent and four percent of glaucoma patients had no non-eye-related hospitalizations or outpatient visits compared with 28% and 16% among the glaucoma negatives, respectively. The annual average time spent hospitalized due to eye- or non-eye-related diagnosis was significantly higher in all glaucoma groups than among persons without glaucoma even after adjusting for age and sex (p < 0.001; Table 3). Travel costs of eye- and non-eye-related outpatient visits were significantly higher in all glaucoma groups than persons without glaucoma even after adjusting for age and sex (p < 0.001). Glaucoma patients had a higher self-reported outpatient health care service use than persons without glaucoma even after adjusting for age and sex (p < 0.001; Fig 2); however, glaucoma patients treated with medication or surgery had lower use of occupational health care than persons without glaucoma (p < 0.001) due to their higher retirement number. Visits to “other doctor” were omitted from the figure due to their low number (average 9 visits / 100 persons / year in the study population). No statistically significant differences were observed within the three glaucoma groups in any of the above-mentioned parameters.

**Table 3:** Mean time spent hospitalized annually per 100 persons adjusted for age and sex.

	Eye-related hospitalization (days; 95% CI)	Non-eye-related hospitalization (days; 95% CI)
Glaucoma, all	14 (12–16)	679 (583–774)
Glaucoma, medication	17 (14–19)	619 (517–721)
Glaucoma, operated	14 (11–18)	742 (552–931)
Glaucoma negatives	2 (2–2)	488 (476–499)

Differences between glaucoma groups and glaucoma negatives were statistically significant (p < 0.001). There were no significant differences within glaucoma groups. Data on hospitalization length were collected during 1999–2011.

CI = confidence interval

Direct mean costs are shown in Table 4 and 95% confidence intervals in S2 Table. All glaucoma groups showed significantly higher direct costs than persons without glaucoma even after adjusting for age and sex (p < 0.001), yet no statistically significant differences were observed within the three glaucoma groups. After adjusting for age and sex, the observed health care expenditure in the total Finnish glaucomatous population was EUR 202 million (non-adjusted EUR 886 million) higher compared with the expected level based on average costs per person in the non-glaucomatous population at the 2019 cost level. The share of eye-related expenses was 12.9% of the age- and sex-adjusted additional expenditure and 2.7% of the non-adjusted additional expenditure among the glaucomatous population. The additional adjusted expenditures were EUR 100 million (non-adjusted EUR 521 million) among glaucoma patients treated with medication and EUR 92 million (non-adjusted EUR 346 million) among operated glaucoma patients. The share of adjusted additional eye-related expenses was 20.9% (non-adjusted 4.1%) for medicated and 7.8% (non-adjusted 2.9%) for operated glaucoma patients. Glaucoma patients who had been operated but did not use glaucoma medication showed two times higher non-eye-related costs in comparison to glaucoma patients with only medical treatment (S3 Table). Most of the direct expenditures came from hospitalizations: 83.4% of adjusted costs (non-adjusted 82.3%) among glaucoma negatives, 78.8% (non-adjusted 91.2%) among glaucoma patients, 81.5% (non-adjusted 89.4%) among glaucoma patients treated with medication, and 73.8% (non-adjusted 90.9%) among operated glaucoma patients. Overall, most of the additional costs among glaucomatous population came from non-eye-related hospitalizations.

**Table 4:** Mean annual direct health care costs in the Finnish population aged 30 years and older at the 2019 cost level.

	Annual costs per person (EUR)												Annual costs in Finland (EUR)
	Hospitalizations		Outpatient visits		Outpatient health care services	Outpatient travels		Total costs^a^		Additional costs (vs. glaucoma negatives)		Population^b^	Total additional costs
	Eye	Non-eye	Eye	Non-eye	All	Eye	Non-eye	Eye	Non-eye	Eye	Non-eye		All
***Non-adjusted costs***													
Glaucoma negatives	22	4,001	16	376	434	2	36	40	4,847			3,067,899	
Glaucoma, all	175	14,915	162	511	722	20	46	357	16,193	318	11,347	75,979	886,240,017
Glaucoma, medication	207	12,436	186	508	729	24	47	417	13,721	378	8,874	56,344	521,259,595
Glaucoma, operated	226	17,866	215	672	846	29	57	471	19,441	431	14,594	22,996	345,523,136
***Adjusted for age and sex***													
Glaucoma negatives	24	4,415	16	379	451	2	36	42	5,281			3,067,899	
Glaucoma, all	152	6,141	209	610	798	24	50	385	7,598	343	2,317	75,979	202,094,791
Glaucoma, medication	178	5,601	209	400	644	25	35	412	6,680	370	1,399	56,344	99,674,677
Glaucoma, operated	154	6,712	177	1,074	1,085	22	79	352	8,950	310	3,669	22,996	91,500,191

All eye- and non-eye-related adjusted and non-adjusted direct annual costs per person were significantly higher in the three glaucoma groups compared with glaucoma negatives (p < 0.001), but there were no significant differences within the three glaucoma groups. 95% confidence intervals are provided in S2 Table.

^a^Total eye costs consist of eye-related hospitalizations, outpatient visits, and outpatient travels during 1999–2011; total non-eye-related costs consist of non-eye-related hospitalizations, outpatient visits, and outpatient travels during 1999–2011 and all outpatient health care services in 2000

^b^Calculated using population weights in the Health 2000 survey

Indirect mean costs due to premature retirement are shown in Table 5 and 95% confidence intervals in S4 Table. A total of 3,801 participants with glaucoma status known reported to have retired by 2011. Among study participants aged 30–64 years, premature retirement was granted to 29 (85.3%) glaucoma patients, 21 (80.8%) glaucoma patients with medication, 7 (70.0%) operated glaucoma patients, and 1572 (29.8%) glaucoma negatives by 2011. There were no statistical differences in personal indirect costs between the three glaucoma groups and glaucoma negatives and within the three glaucoma groups. However, at the population level, glaucoma was associated with a total additional expenditure of EUR 71 million per year in comparison to glaucoma negatives at the 2019 cost level. The additional expenditures were EUR 41 million among glaucoma patients treated with medication and EUR 63 million among operated glaucoma patients. Productivity losses comprised majority (70.9%) of the total indirect expenditures in all groups.

**Table 5:** Mean indirect costs in the Finnish population aged 30–64 years at the 2019 cost level.

	Costs per person retired prematurely (EUR)				Annual costs per person retired prematurely (EUR)^a^			Annual costs in Finland (EUR)^a^
	Premature retirement	Productivity loss	Total costs	Additional costs (vs. glaucoma negatives)	Total costs	Additional costs (vs. glaucoma negatives)	Population^b^	Total additional costs
Glaucoma negatives	154,185	376,151	530,336		16,268		2,415,553	
Glaucoma, all	194,823	475,294	670,118	139,782	20,556	4,288	16,613	71,233,046
Glaucoma, medication	184,947	451,198	636,145	105,809	19,514	3,246	12,687	41,177,951
Glaucoma, operated	276,467	674,473	950,941	420,605	29,170	12,902	4,902	63,245,527

No statistical differences were observed in personal indirect costs between the three glaucoma groups and glaucoma negatives and within the three glaucoma groups. Data were collected during 1999–2011. 95% confidence intervals are provided in S4 Table.

^a^Annual costs calculated by dividing costs per person by the average years expected to work in a lifetime in Finland (32.6 years in 2011) [24]

^b^Calculated using population weights in the Health 2000 survey

After adjusting for age, sex, and non-eye-related co-morbidities (S5 Table), glaucoma or its treatment did not show statistically significant association with total direct costs compared with glaucoma negatives. When sex and non-eye-related co-morbidities were set constant and age at the average of the glaucomatous population in Finland (71.9 years), the mean annual total direct costs were EUR 46,746 (95% confidence interval [CI] 27,470–66,022) for a glaucoma patient, EUR 43,591 (95% CI 23,985–63,196) for a glaucoma patient with medical treatment, and EUR 54,721 (95% CI 28,570–80,872) for an operated glaucoma patient at the 2019 cost level. In a model that also included eye-related co-morbidities (unoperated cataract, retinal degeneration, visual impairment), visual impairment showed third strongest impact on total direct costs after Parkinson’s disease and psychiatric disorders.

Total indirect costs adjusted for age, sex, and non-eye-related are shown in Table 6. Only operated glaucoma showed statistically significant association with total indirect costs compared with glaucoma negatives after adjusting for these predictors (additional indirect costs EUR 23,015; p = 0.019). When sex and non-eye-related co-morbidities were set constant and age at the average of the glaucomatous population below age of 65 years in Finland (55.3 years), the mean annual total indirect costs were EUR 33,718 (95% CI 20,857–46,578) for a glaucoma patient, EUR 33,974 (95% CI 19,168–47,780) for a glaucoma patient with medical treatment, and EUR 49,204 (95% CI 21,159–77,249) for an operated glaucoma patient at the 2019 cost level. In a model that also included eye-related co-morbidities, visual impairment showed strongest impact on total indirect costs of all included predictors.

**Table 6:** Multivariable regression analysis examining the impact of glaucoma, age, sex, and non-eye-related co-morbidities on total annual indirect costs in population aged 30–64 years at the 2019 cost level.

	B coefficient	Marginal mean (EUR)	Marginal mean contrast (EUR)	P value		B coefficient	Marginal mean (EUR)	Marginal mean contrast (EUR)	P value		B coefficient	Marginal mean (EUR)	Marginal mean contrast (EUR)	P value
Constant	12.980			< 0.001	Constant	12.984			< 0.001	Constant	12.954			< 0.001
Age	–0.006			0.10	Age	–0.006			0.10	Age	–0.005			0.13
Male sex	0.091	31,027	2,695	0.023	Male sex	0.091	31,184	2,722	0.022	Male sex	0.088	37,503	3,142	0.030
Glaucoma, all	0.257	33,718	7,647	0.10	Glaucoma, medication	0.263	33,974	7,849	0.15	Glaucoma, operated	0.631	49,204	23,015	0.019
Heart disease	0.248	27,328	6,006	< 0.001	Heart disease	0.251	27,495	6,096	< 0.001	Heart disease	0.251	33,133	7,354	< 0.001
Pulmonary disease	0.097	25,333	2,332	0.037	Pulmonary disease	0.094	25,421	2,277	0.043	Pulmonary disease	0.094	30,628	2,739	0.043
Vascular disease	0.094	25,296	2,261	0.09	Vascular disease	0.097	25,458	2,347	0.08	Vascular disease	0.091	30,583	2,654	0.10
Musculoskeletal condition	0.095	25,309	2,285	0.036	Musculoskeletal condition	0.094	25,422	2,280	0.038	Musculoskeletal condition	0.096	30,658	2,797	0.033
Hypertension	0.012	24,281	282	0.79	Hypertension	0.008	24,356	199	0.85	Hypertension	0.008	29,346	240	0.85
Diabetes	0.171	26,300	4,144	0.021	Diabetes	0.175	26,475	4,252	0.018	Diabetes	0.181	31,988	5,286	0.016
Psychiatric disorder	0.446	30,163	10,846	< 0.001	Psychiatric disorder	0.441	30,240	10,784	< 0.001	Psychiatric disorder	0.453	36,651	13,345	< 0.001
Parkinson’s disease	0.152	26,050	3,682	0.52	Parkinson’s disease	0.154	26,199	3,742	0.51	Parkinson’s disease	0.154	31,560	4,495	0.52
Cancer	0.150	26,025	3,635	0.13	Cancer	0.151	26,158	3,666	0.13	Cancer	0.147	31,462	4,312	0.14

Tweedie distribution using gamma with log link scale response was applied to the model. The analysis was based on participants with information available for all predictors (*n* = 1688–1710). The age was standardized for the average age of glaucomatous population in Finland under 65 years of age (55.3 years) for the marginal means and contrasts. Marginal mean contrasts equal the difference between those with a medical condition (or of male sex) and those without a medical condition (or of female sex) standardized for all other factors. Statistical significance was calculated for both the B coefficients and marginal mean contrasts.

**Fig 3:**
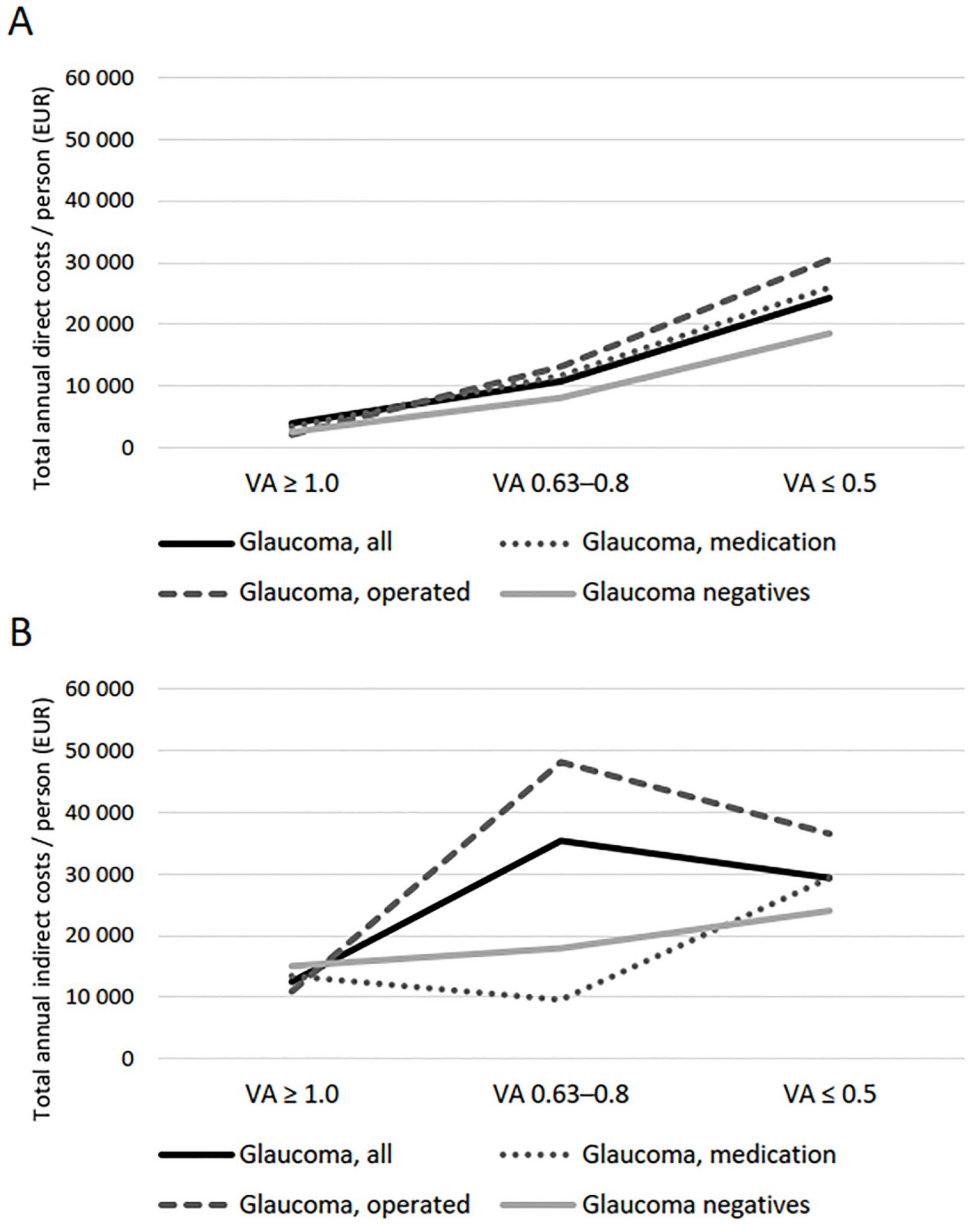
Association between average distance visual acuity (VA) and total annual direct costs (A) and indirect costs (B) among glaucoma patients and glaucoma negatives at the 2019 cost level. Direct costs were evaluated in population aged 30 years and older, and indirect costs in population aged 30–64 years.

The association between distance vision and both direct and indirect costs is illustrated in Fig 3. The two lowest vision groups were combined due to low number of glaucoma patients under 65 years of age in these groups. A strong negative association between vision and costs was observed regardless of whether a person has glaucoma or not: correlation coefficients in the studied groups ranged from -0.24 to -0.36 regarding direct costs and from -0.16 to -0.58 regarding indirect costs. Although both direct and indirect cost appeared to be higher among glaucoma patients than negatives, no statistically significant differences were observed.

## Discussion

To our knowledge, this is the first cost-of-illness study of glaucoma to report both direct and indirect costs associated with the disease based on nationally representative data. The comprehensive data allowed us to include eye- and non-eye-related treatments, as well as to compare glaucoma with other co-morbidities. Here we show that glaucoma is associated with a high economic burden on the society. The major proportion of the costs is not directly caused by treatment of glaucoma, but rather the increased use of non-eye-related health services, as well as loss of productivity. In addition, different treatment options for glaucoma show noticeable differences in costs and resource use.

We calculated age- and sex-adjusted costs because glaucoma patients are in average 20 years older than persons without glaucoma. In 2019, the expenditures of health care in Finland were EUR 23.4 billion in total [31]. In the present study, the adjusted direct additional expenditures associated with glaucoma corresponded to 0.86% (EUR 202,094,791) of this cost. The prevalence of glaucoma in Finnish adult population is approximately 2.6% [2], and this figure is likely to increase due to the rapid ageing of the Finnish population. Therefore, the direct costs of glaucoma can be considered significant, and this economic burden is likely to increase in the future with increasing life expectancy and shifting in age distribution in Finland and other developed countries.

While glaucoma care has been organized in different ways around the world, glaucoma is globally considered a major burden for health care resources. In the US, the annual direct medical costs of glaucoma were estimated to be USD 2.9 billion in 2004 [13]. In Australia, the annual direct eye-related costs of glaucoma were estimated to be AUD 144.2 million in 2004 [14]. In both countries, the direct medical costs of glaucoma corresponded to 8% of total medical costs of visual disorders [13, 14]. Furthermore, the costs of glaucoma are usually considered underestimated due to the high percentage of undiagnosed glaucoma [32, 33].

Despite the economic implications of glaucoma, few studies have provided nationwide estimations of all direct and indirect costs of the disease. In 1990 in the UK, the direct medical costs associated with glaucoma were GBP 61 million, direct non-medical costs GBP 25 million among visually impaired, and indirect costs GBP 45 million [34]. In a more recent study in Nigeria, Adio and Onua reported an annual direct and indirect loss of USD 1,265 per person for treatment of glaucoma, resulting in a total expenditure of USD 4,095,000 [35]. However, both studies only included costs related to glaucoma treatment, which explains why the average costs are lower than in our study. Finally, in a review by Dirani et al., they created a prediction model on primary open-angle glaucoma in Australia that during 2005–2025 direct health system costs will increase from AUD 355 million to AUD 784 million and total costs (direct and indirect) from AUD 1.9 billion to AUD 4.3 billion [36].

Medication represents the major cost of glaucoma treatment. In the US, the cost of glaucoma care for Medicare beneficiaries was USD 748 million in 2009 [15]. In Sweden and France, the respective annual costs of glaucoma treatment were EUR 531 and EUR 390 per patient, with medication costs comprising approximately half of the total costs [9]. In Denmark, the annual treatment cost was EUR 305 per glaucoma patient under their initial regimen, of which 57% was accounted by glaucoma drugs [11]. In Finland, the total cost of glaucoma medication was EUR 25.5 million in 2011 with an average of EUR 352 per patient [37]. When adding the direct eye-related treatment costs in our study (EUR 393 per patient), the average annual glaucoma treatment cost per medicated glaucoma patient at 2019-level would be EUR 745, 47% consisting of medication costs, which is within the range of previous glaucoma resource utilization studies. The high costs associated with glaucoma medicine are likely due to the increased consumption of anti-glaucoma drugs in recent decades and the use of newer and more expensive drugs [36]. Furthermore, the severity of glaucoma has been reported to increase the direct costs of its treatment [38, 39].

There has not been definitive conclusion on whether medical or surgical treatment of glaucoma is more cost-effective [9]. In our study, operated glaucoma patients showed higher use of outpatient care and hospitalization than medicated patients. Although this difference was not statistically significant, it becomes particularly noticeable when costs are considered: even after adjusting for age and sex, the annual total direct costs are EUR 2,210 (31.2%) higher for an operated patient than medicated patient. Still, if the estimated drug costs [37] are added to the expenditures associated with medicated glaucoma, the expenditures for medicated glaucoma patients are higher than reported. The annual indirect costs for an operated patient are EUR 9,656 (49.5%) higher compared with a medicated patient. Patients needing glaucoma surgery are in general more often unable to take care of their medication due to their co-morbidities. This is one of the possible explanations why glaucoma patients only treated with surgery showed higher total direct costs than glaucoma patients only treated with medication. It is also important to remember that glaucoma surgery is in many cases the last option to prevent the progression of glaucoma and consequent visual loss, both of which are associated with additional direct and indirect costs.

Despite the role of treatment in the economic burden of glaucoma observed in previous studies, in our study, majority of the direct health care costs came from non-eye-related services. We also observed a significant increase in the average time spent hospitalized among glaucoma patients in comparison to non-glaucomatous population. This is most likely related to the irreversible vision loss associated with glaucoma and its progression. The severity of visual impairment increases the resource consumption and intensity of care likely due to the increased risk of falls, accidents, and injuries associated with decreased vision [40]. Indeed, glaucoma patients have been reported increased risk of falls and other accidents, which contribute to significant amount of bed days with an economic and operational impact on the hospitals [41–43]. Vision loss is associated with high economic impact [44], and the costs among blinded patients can be twice the amount among patients with normal vision [40]. Also, we observed a strong relationship between decreasing vision and both increasing direct and indirect costs regardless of glaucoma status. Therefore, the role of early intervention in glaucoma care to prevent the progression of visual impairment is vital in alleviating the economic burden of the disease to the society, as well as the detrimental effect on quality of life, independence, and social activity of the patient [17, 45, 46]. In addition, the impact of vision on the societal costs calls for further research.

The indirect costs associated with glaucoma are also considerable. The Finnish gross domestic product was EUR 239.9 billion in 2019 according to the Statistics Finland -database. Additional productivity losses caused by glaucoma alone corresponded to 0.03% (EUR 71,233,046) of the product that year. Loss of productivity among glaucoma patients is likely contributable to vision loss associated with the disease, as visual impairment is associated with nursing home admission, falls, injuries, accidents, and femur fracturs, all of which can lead to work invalidity [40]. Visual impairment and blindness are regarded as major causes of productivity losses worldwide [47]. Therefore, by preventing the progression of vision loss due to glaucoma with early diagnosis and prompt and adhered treatment, significant economic losses could be averted.

Both direct and indirect glaucoma costs showed strong dependency on vision and other co-morbidities. These factors are associated either directly with glaucoma or indirectly through ageing [48–50], which likely explains this effect. However, the indirect additional costs of operated glaucoma are significant even after adjusting for these co-morbidities, which implies the severity and specific surgical indications of the operated glaucoma patients.

The strengths of our study include the representative sample of the Finnish adult population, the multiple data sources, and the long follow-up period that increase the validity and reliability of the results. The Health 2000 Survey addressed public health issue more broadly than national surveys do on average. The survey sample represents the population particularly well due to the comprehensive sampling design and the high participation rate. This allowed us to include a sample of glaucoma patients and negatives at national level rather than from clinical settings. The data design of the national health survey reduces the impact of potential confounding factors, which was further reduced by controlling the co-morbidities and other confounding factors using multivariable modelling. In addition, the applied weighting scheme improves the applicability to population level. Our prevalence-based bottom-up approach aids to avoid the misallocation of costs, which is more likely to occur in top-down approach [51]. Although prevalence-based approach may not accurately quantify the long-term consequences of the study condition leading to underestimation of costs [51], our long, 13-year follow-up time should alleviate the potential bias associated with this approach.

Our study also has limitations that need to be addressed. While our use of multiple data sources can be regarded as a major strength, it also can produce difficulties in processing and integrating data as its availability varied between sources. The time differences between key inputs should be considered, as the data were collected during a 13-year follow-up time during 1999–2011 and the costs were converted to the 2019 level. We could not differentiate eye- and non-eye-related self-reported outpatient health care service visits. While the share of ophthalmologist visits in health centers should be small, the share among private practitioners can be higher, therefore causing bias. We were not able include laboratory costs in private health care in the calculations due to the classified nature of the data. However, laboratory costs are included in public health care unit costs, which should alleviate this deficiency in total cost analyses. We were also unable to include the costs of care outside the health system as well as non-health care costs, for example, those caused by social services, childcare, and housekeeping. Drugs and prescriptions were also not included in the cost analyses, although we discussed their share based on the medication cost estimations by Parkkari and co-workers [37]. While the cost of disability pensions and premature retirement were included, productivity losses might be underestimated because we were not able to get data on sick leaves. Despite this, the estimated costs in this study are generalizable to the Finnish adult population or to a similar setting in terms of population age structure and financial support system from government, such as all Nordic countries and several European countries. Glaucoma classifications were based on register data on observations made by a private ophthalmologist, which can cause biases: for example, high intraocular pressure may have been diagnosed as glaucoma, even though it may not have been the case.

In conclusion, we report annual direct and indirect additional expenditures of EUR 202,094,791 and EUR 71,233,046 among glaucomatous population in Finland. Therefore, glaucoma is a significant economic burden on the health care and society. Majority of the direct expenses come from non-eye-related hospitalizations, and productivity losses comprise most of the indirect expenses. The need for expensive hospitalization is most likely contributable to the progressing vision loss and consequent increase in risk of injuries and accidents among glaucoma patients. The high age and consequent increase in co-morbidities among glaucoma patients are also contributable factors to the additional costs of glaucoma. Moreover, different glaucoma treatments show substantial variability in costs and resource use, most probably due to the uneven distribution of co-morbidities. Given the limited resources available to health care providers, early-stage interventions to prevent glaucoma progression as well as allocating sufficient resources to ophthalmic care are a necessity to avoid economic challenges in the future as the population ages. The increased allocation may pay itself off multiple times with the future savings. Further research in other countries is necessary to address the economic implications of glaucoma in the big picture to confirm our results and to help the prioritizing of health care resources.

## Supporting information

S1 Appendix CHEERS 2022 guidelines followed in this study. (DOCX)

S1 Table Direct and indirect costs in Finland in 2011 and 2019. (DOCX)

S2 Table Mean annual direct health care costs with 95% confidence intervals (CIs) in the Finnish population aged 30 years and older at the 2019 cost level. (DOCX)

S3 Table Mean non-adjusted direct health care costs in glaucoma patients with different treatments at the 2019 cost level. (DOCX)

S4 Table Mean indirect costs with 95% confidence intervals (CIs) in the Finnish population aged 30–64 years at the 2019 cost level. (DOCX)

S5 Table Multivariable regression analysis examining the impact of glaucoma, age, sex, and non-eye-related co-morbidities on total annual direct health care costs in population aged 30 years and older at the 2019 cost level. (DOCX)
