# Correction: Price tag of glaucoma care is minor compared with the total direct and indirect costs of glaucoma: Results from nationwide survey and register data

**DOI:** 10.1371/journal.pone.0318723

**Published:** 2025-01-30

**Authors:** Petri K. M. Purola, Joonas Taipale, Saku Väätäinen, Mika Harju, Seppo V. P. Koskinen, Hannu M. T. Uusitalo

In the Result subsection of the Abstract, there are errors in the paragraph. The correct paragraph is: The annual additional total direct costs were EUR 2,658/glaucoma patient, EUR 1,768/glaucoma patient with medication, and EUR 3,975/operated glaucoma patient compared with persons without glaucoma. The respective additional total indirect costs were EUR 4,035, EUR 3,054, and EUR 12,141 per year. In total, the additional annual direct and indirect expenditures associated with glaucoma in Finland were EUR 202 million (0.86% of total expenditures of health care) and EUR 67 million (0.03% of the Finnish gross domestic product) arising mainly from non-eye-related hospitalizations and productivity losses, respectively.

In the Introduction, there is an error in the third sentence of the first paragraph. The correct sentence is: In Finland, there are over 80,000 glaucoma patients, of which approximately 8% are visually impaired with visual acuity (VA) lower than 0.3 (Snellen decimals) [2,3].

In the Cost analysis subsection of the Materials and methods, there is an error in the ninth sentence of the first paragraph. The correct sentence is: The unit costs do not include the customer fees as our focus was on societal costs.

In the Results, there is an error in the fourth and fifth sentences of the third paragraph. The correct sentences are: The share of eye-related expenses was 12.8% of the age- and sex-adjusted additional expenditure and 2.7% of the non-adjusted additional expenditure among the glaucomatous population. The additional adjusted expenditures were EUR 100 million (non-adjusted EUR 521 million) among glaucoma patients treated with medication and EUR 91 million (non-adjusted EUR 345 million) among operated glaucoma patients.

In the Results, there is an error in the eighth sentence of the third paragraph. The correct sentence is: Most of the direct expenditures came from hospitalizations: 83.4% of adjusted costs (non-adjusted 82.3%) among glaucoma negatives, 78.9% (non-adjusted 91.2%) among glaucoma patients, 81.5% (non-adjusted 89.5%) among glaucoma patients treated with medication, and 73.9% (non-adjusted 90.9%) among operated glaucoma patients.

In the Results, there is an error in the fifth to seventh sentences of the fourth paragraph. The correct sentences are: However, at the population level, glaucoma was associated with a total additional expenditure of EUR 67 million per year in comparison to glaucoma negatives at the 2019 cost level. The additional expenditures were EUR 38 million among glaucoma patients treated with medication and EUR 59 million among operated glaucoma patients. Productivity losses comprised majority (69.1%) of the total indirect expenditures in all groups.

In the Results, there is an error in the second and third sentences of the sixth paragraph. The correct sentences are: Only operated glaucoma showed statistically significant association with total indirect costs compared with glaucoma negatives after adjusting for these predictors (additional indirect costs EUR21,658; p = 0.019). When sex and non-eye-related co-morbidities were set constant and age at the average of the glaucomatous population below age of 65 years in Finland (55.3 years), the mean annual total indirect costs were EUR 31,730 (95% CI 19,628–43,832) for a glaucoma patient, EUR 31,971 (95% CI 18,038–45,904) for a glaucoma patient with medical treatment, and EUR 46,303 (95% CI 19,912–72,694) for an operated glaucoma patient at the 2019 cost level.

In the Results, there is an error in the third sentence of the seventh paragraph. The correct sentence is: A strong negative association between vision and costs was observed regardless of whether a person has glaucoma or not: correlation coefficients in the studied groups ranged from -0.24 to -0.36 regarding direct costs and from -0.16 to -0.67 regarding indirect costs.

In the Discussion, there is an error in the third sentence of the second paragraph. The correct sentence is: In the present study, the adjusted direct additional expenditures associated with glaucoma corresponded to 0.86% (EUR 201,931,493) of this cost.

In the Discussion, there is an error in the sixth sentence of the fifth paragraph. The correct sentence is: When adding the direct eye-related treatment costs in our study (EUR 387 per patient), the average annual glaucoma treatment cost per medicated glaucoma patient at 2019-level would be EUR 739, 48% consisting of medication costs, which is within the range of previous glaucoma resource utilization studies.

In the Discussion, there is an error in the third sentence of the sixth paragraph. The correct sentence is: Although this difference was not statistically significant, it becomes particularly noticeable when costs are considered: even after adjusting for age and sex, the annual total direct costs are EUR 2,207 (31.1%) higher for an operated patient than medicated patient.

In the Discussion, there is an error in the fifth sentence of the sixth paragraph. The correct sentence is: The annual indirect costs for an operated patient are EUR 9,087 (49.5%) higher compared with a medicated patient.

In the Discussion, there is an error in the third sentence of the eighth paragraph. The correct sentence is: Additional productivity losses caused by glaucoma alone corresponded to 0.03% (EUR67,032,633) of the product that year.

In the Discussion, there is an error in the first sentence of the 12^th^ paragraph. The correct sentence is: In conclusion, we report annual direct and indirect additional expenditures of EUR 201,931,493 and EUR 67,032,633 among glaucomatous population in Finland.

The Tables 4 to 6 are incorrect. Please see the correct Tables 4 to 6 here.

**Table 4:** Mean annual direct health care costs in the Finnish population aged 30 years and older at the 2019 cost level.

	Annual costs per person (EUR)												Annual costs in Finland (EUR)
	Hospitalizations		Outpatient visits		Outpatient health care services	Outpatient travels		Total costs^a^		Additional costs (vs. glaucoma negatives)		Population^b^	Total additional costs
	Eye	Non-eye	Eye	Non-eye	All	Eye	Non-eye	Eye	Non-eye	Eye	Non-eye		All
***Non-adjusted costs***													
Glaucoma negatives	22	4,001	16	376	434	2	34	39	4,845			3,067,899	
Glaucoma, all	175	14,915	162	511	722	19	43	356	16,191	317	11,346	75,979	886,109,578
Glaucoma, medication	207	12,436	186	508	729	22	44	416	13,718	376	8,873	56,344	521,148,787
Glaucoma, operated	226	17,866	215	672	846	27	53	469	19,437	430	14,593	22,996	345,456,778
***Adjusted for age and sex***													
Glaucoma negatives	24	4,415	16	379	451	2	34	42	5,279			3,067,899	
Glaucoma, all	152	6,141	209	610	798	23	47	383	7,595	341	2,316	75,979	201,931,493
Glaucoma, medication	178	5,601	209	400	644	24	33	411	6,678	369	1,399	56,344	99,601,029
Glaucoma, operated	154	6,712	177	1,074	1,085	20	74	351	8,945	309	3,666	22,996	91,413,338

All eye- and non-eye-related adjusted and non-adjusted direct annual costs per person were significantly higher in the three glaucoma groups compared with glaucoma negatives (p < 0.001), but there were no significant differences within the three glaucoma groups. 95% confidence intervals are provided in S2 Table.

^a^Total eye costs consist of eye-related hospitalizations, outpatient visits, and outpatient travels during 1999–2011; total non-eye-related costs consist of non-eye-related hospitalizations, outpatient visits, and outpatient travels during 1999–2011 and all outpatient health care services in 2000

^b^Calculated using population weights in the Health 2000 survey

**Table 5:** Mean indirect costs in the Finnish population aged 30–64 years at the 2019 cost level.

	Costs per person retired prematurely (EUR)				Annual costs per person retired prematurely (EUR)^a^			Annual costs in Finland (EUR)^a^
	Premature retirement	Productivity loss	Total costs	Additional costs (vs. glaucoma negatives)	Total costs	Additional costs (vs. glaucoma negatives)	Population^b^	Total additional costs
Glaucoma negatives	154,185	344,879	499,063		15,309		2,415,553	
Glaucoma, all	194,823	435,779	630,603	131,539	19,344	4,035	16,613	67,032,633
Glaucoma, medication	184,947	413,687	598,633	99,570	18,363	3,054	12,687	38,749,803
Glaucoma, operated	276,467	618,399	894,866	395,803	27,450	12,141	4,902	59,516,116

No statistical differences were observed in personal indirect costs between the three glaucoma groups and glaucoma negatives and within the three glaucoma groups. Data were collected during 1999–2011. 95% confidence intervals are provided in S4 Table.

^a^Annual costs calculated by dividing costs per person by the average years expected to work in a lifetime in Finland (32.6 years in 2011) [24]

^b^Calculated using population weights in the Health 2000 survey

**Table 6:** Multivariable regression analysis examining the impact of glaucoma, age, sex, and non-eye-related co-morbidities on total annual indirect costs in population aged 30–64 years at the 2019 cost level.

	B coefficient	Marginal mean (EUR)	Marginal mean contrast (EUR)	P value		B coefficient	Marginal mean (EUR)	Marginal mean contrast (EUR)	P value		B coefficient	Marginal mean (EUR)	Marginal mean contrast (EUR)	P value
Constant	12.919			< 0.001	Constant	12.923			< 0.001	Constant	12.893			< 0.001
Age	–0.006			0.10		Age	–0.006			0.10	Age	–0.005			0.13
Male sex	0.091	29,198	2,536	0.023	Male sex	0.091	29,345	2,561	0.022	Male sex	0.088	35,291	2,957	0.030
Glaucoma, all	0.257	31,730	7,196	0.10	Glaucoma, medication	0.263	31,971	7,386	0.15	Glaucoma, operated	0.631	46,303	21,658	0.019
Heart disease	0.248	25,717	5,652	< 0.001	Heart disease	0.251	25,873	5,737	< 0.001	Heart disease	0.251	31,180	6,920	< 0.001
Pulmonary disease	0.097	23,839	2,194	0.037	Pulmonary disease	0.094	23,922	2,143	0.043	Pulmonary disease	0.094	28,822	2,577	0.043
Vascular disease	0.094	23,804	2,127	0.09	Vascular disease	0.097	23,957	2,209	0.08	Vascular disease	0.091	28,780	2,497	0.10
Musculoskeletal condition	0.095	23,816	2,150	0.036	Musculoskeletal condition	0.094	23,923	2,146	0.038	Musculoskeletal condition	0.096	28,850	2,632	0.033
Hypertension	0.012	22,849	266	0.79	Hypertension	0.008	22,919	188	0.85	Hypertension	0.008	27,616	226	0.85
Diabetes	0.171	24,749	3,900	0.021	Diabetes	0.175	24,914	4,001	0.018	Diabetes	0.181	30,102	4,974	0.016
Psychiatric disorder	0.446	28,385	10,206	< 0.001	Psychiatric disorder	0.441	28,457	10,148	< 0.001	Psychiatric disorder	0.453	34,490	12,558	< 0.001
Parkinson’s disease	0.152	24,514	3,465	0.52	Parkinson’s disease	0.154	24,654	3,521	0.51	Parkinson’s disease	0.154	29,699	4,230	0.52
Cancer	0.150	24,490	3,421	0.13	Cancer	0.151	24,616	3,450	0.13	Cancer	0.147	29,606	4,058	0.14

Tweedie distribution using gamma with log link scale response was applied to the model. The analysis was based on participants with information available for all predictors (*n* = 1688–1710). The age was standardized for the average age of glaucomatous population in Finland under 65 years of age (55.3 years) for the marginal means and contrasts. Marginal mean contrasts equal the difference between those with a medical condition (or of male sex) and those without a medical condition (or of female sex) standardized for all other factors. Statistical significance was calculated for both the B coefficients and marginal mean contrast.

In Fig 3, the diagram B is incorrect. Please see the correct Fig 3 here.

**Fig 3:**
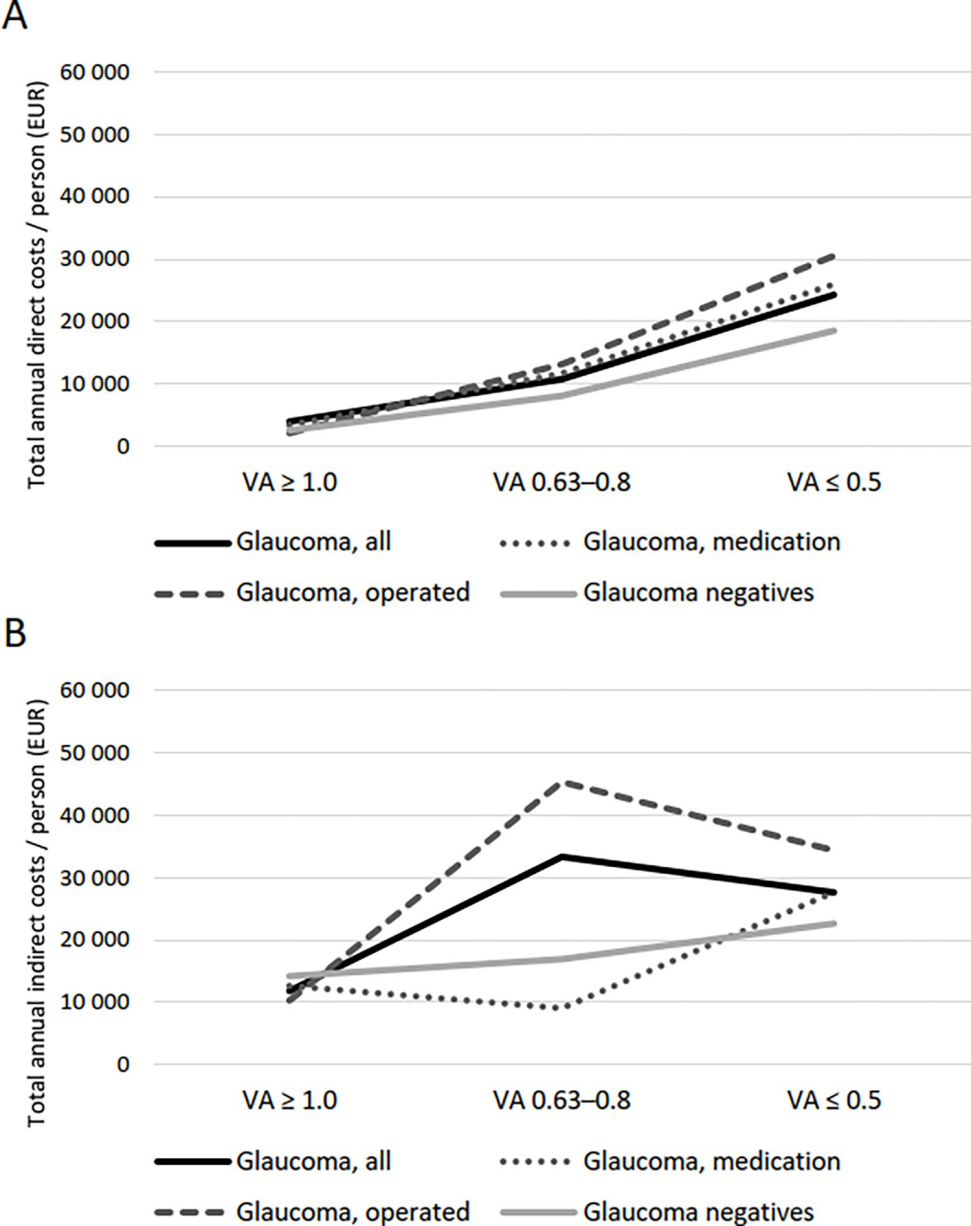
**Association between average distance visual acuity (VA) and total annual direct costs (A) and indirect costs (B) among glaucoma patients and glaucoma negatives at the 2019 cost level**. Direct costs were evaluated in population aged 30 years and older, and indirect costs in population aged 30–64 years.

The S1, S2 and S4 Tables are incorrect. Please view the correct S1, S2 and S4 Tables below.

## Supporting information

S1 Table Direct and indirect costs in Finland in 2011 and 2019. (DOCX)

S2 Table Mean annual direct health care costs with 95% confidence intervals (CIs) in the Finnish population aged 30 years and older at the 2019 cost level. (DOCX)

S4 Table Mean indirect costs with 95% confidence intervals (CIs) in the Finnish population aged 30–64 years at the 2019 cost level. (DOCX)
