# Supplementary material for: Price tag of glaucoma care is minor compared with the total direct and indirect costs of glaucoma: Results from nationwide survey and register data
Source: PLoS One. 2023 Dec 20;18(12):e0295523. doi: 10.1371/journal.pone.0295523 (PMC10732367; doi:10.1371/journal.pone.0295523)
Supplement: S1 Appendix — (DOCX) [file pone.0295523.s001.docx]

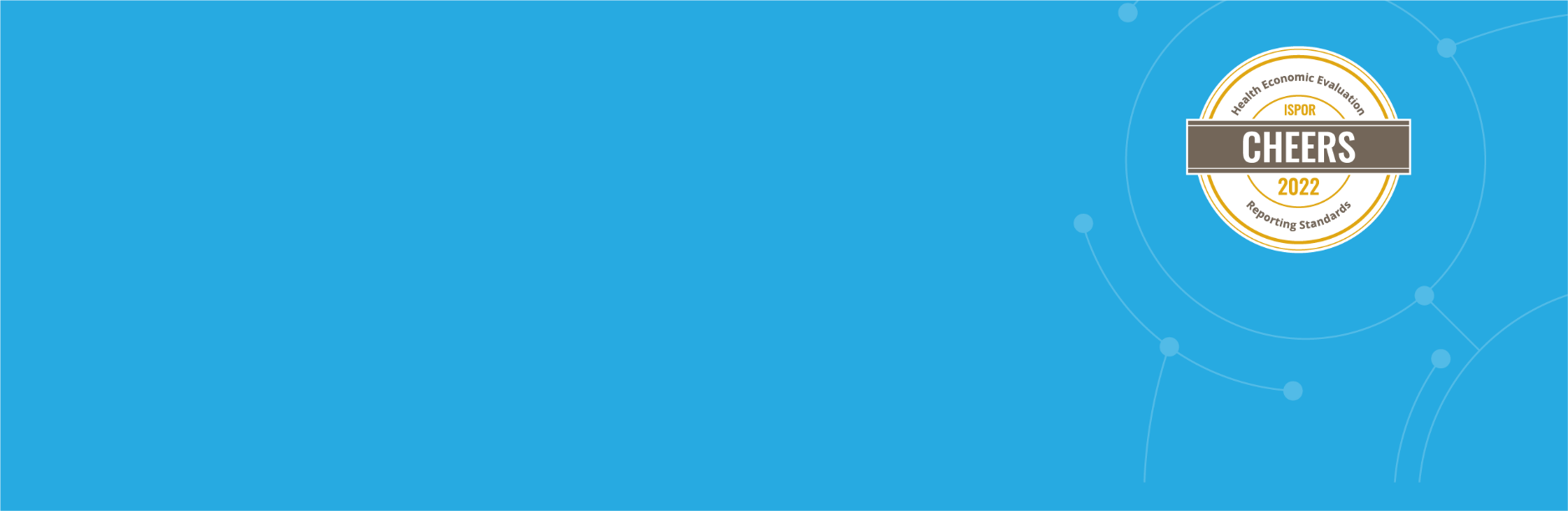


**Consolidated Health Economic Evaluation Reporting Standards
(CHEERS) 2022 Checklist**

The CHEERS 2022 statement replaces the 2013 CHEERS statement, which should no longer
be used. The CHEERS 2022 checklist contains 28 items with accompanying descriptions.
Checklist users should indicate the section of the manuscript where relevant information
can be found. The authors recommend using a section heading with a paragraph number.
If an item does not apply to a particular economic evaluation, checklist users are encouraged
to report “Not Applicable.” If information is otherwise not reported, checklist users are encouraged to

write, “Not Reported.” Users should avoid the term “Not Conducted” as CHEERS is intended to guide and

capture reporting. Additional information on CHEERS 2022 can be found here.

**Title**

**1. Title**

Identify the study as an economic evaluation and specify the interventions being compared.

Price tag of glaucoma care is minor compared with the total direct and indirect costs of glaucoma: results from nationwide survey and register data

**Abstract**

**2. Abstract**

Provide a structured summary that highlights context, key methods, results, and alternative analyses.

See Abstract.

**Introduction**

**3. Introduction: Background and Objectives**

Give the context for the study, the study question, and its practical relevance for decision making in policy or practice.

See Introduction.

**Methods**

**4. Health economic analysis plan**

Indicate whether a health economic analysis plan was developed and where available.

Not Applicable.

**5. Study population**

Describe characteristics of the study population (such as age range, demographics, socioeconomic, or clinical characteristics).

The representative sample of the Finnish adult population was selected for the Health 2000 Survey by utilizing a probability-clustered sampling and weighting scheme. The sample included 8,028 subjects (55% women) aged 30 years and older (mean age 54 years), and the unweighted participation rate was 93%. Details of the sample in this study are shown in Table 2.

**6. Setting and location**

Provide relevant contextual information that may influence findings.

The target population of the Health 2000 Survey consisted of individuals aged 18 or over and living in mainland Finland. In addition to the household population, people living in institutions were included. The main survey was carried out in the population aged 30 or over, and a study of young adults was focused on people aged 18–29. Furthermore, in order to obtain a sufficient number of observations from the oldest age cohorts in the main survey, people aged 80 or over were oversampled using a double sampling fraction.

**7. Comparators**

Describe the interventions or strategies being compared and why chosen.

Studied interventions included medical and surgical treatments of glaucoma, as well as a general comparison between all glaucoma patients and persons without glaucoma. Glaucoma patients were selected according to various register data, which are shown in detail in Table 1.

**8. Perspective**

State the perspective(s) adopted by the study and why chosen.

There is a need for a comprehensive picture of the economic burden of glaucoma and its treatments including all eye- and non-eye-related direct and indirect costs associated with the disease—for example, hospitalizations due to falls and injuries. More population-wide studies are also required to corroborate the previous findings and to provide accurate estimates of the costs in different nationwide settings. Furthermore, the use of multiple data sources, such as national surveys and registers, is uncommon, even though it could provide more accurate estimates on the use of health care services and both direct and indirect costs. Therefore, the aim of our study was to evaluate the economic impact of glaucoma on the Finnish society and compare the effect of its treatments by combining the data of a nationwide health examination survey and national health registers and including both direct and indirect costs.

**9. Time horizon**

State the time horizon for the study and why appropriate.

The 13-year follow-up lasted from 1.1.1999 to 31.12.2011. This time set was chosen based on the availability of various register data. Furthermore, the long follow-up duration should alleviate the potential biases of long-term consequences in our prevalence-based approach.

**10. Discount rate**

Report the discount rate(s) and reason chosen.

Not Applicable.

**11. Selection of outcomes**

Describe what outcomes were used as the measure(s) of benefit(s) and harm(s).

Not Applicable, as this is a register-based study.

**12. Measurement of outcomes**

Describe how outcomes used to capture benefit(s) and harm(s) were measured.

Not Applicable, as this is a register-based study.

**13. Valuation of outcomes**

Describe the population and methods used to measure and value outcomes.

See Methods: Study design, data, and population, and Methods: Cost analysis.

**14. Measurement and valuation of resources and costs**

Describe how costs were valued.

See Methods: Cost analysis.

**15. Currency, price date, and conversion**

Report the dates of the estimated resource quantities and unit costs, plus the currency and year of conversion.

See S1 Table.

**16. Rationale and description of model**

If modeling is used, describe in detail and why used. Report if the model is publicly available and where it can be accessed.

Not Applicable.

**17. Analytics and assumptions**

Describe any methods for analyzing or statistically transforming data, any extrapolation methods, and approaches for validating any model used.

Because the cost data were right-skewed and the proportion of participants with zero costs was under 20%, we applied Tweedie distribution using gamma with log link scale response for multivariable regression analyses.

**18. Characterizing heterogeneity**

Describe any methods used for estimating how the results of the study vary for subgroups.

We calculated both non-adjusted and age-sex-adjusted results to account for the differences between glaucoma patients and non-glaucomatous population, as well as between operated and medically treated glaucoma patients.

**19. Characterizing distributional effects**

Describe how impacts are distributed across different individuals or adjustments made to reflect priority populations.

Not Applicable.

**20. Characterizing uncertainty**

Describe methods to characterize any sources of uncertainty in the analysis.

To measure the uncertainty, we calculated 95% confidence intervals for all results.

**21. Approach to engagement with patients and others affected by the study**

Describe any approaches to engage patients or service recipients, the general public, communities, or stakeholders (eg, clinicians or payers) in the design of the study.

Not Applicable.

**Results**

**22. Study parameters**

Report all analytic inputs (eg, values, ranges, references) including uncertainty or distributional assumptions.

See Results, Figures 1–3, Tables 3–6, and Tables S2–S3.

**23. Summary of main results**

Report the mean values for the main categories of costs and outcomes of interest and summarize them in the most appropriate overall measure.

See Results and Tables 3–6.

**24. Effect of uncertainty**

Describe how uncertainty about analytic judgments, inputs, or projections affects findings. Report the effect of choice of discount rate and time horizon, if applicable.

Population weights calculated by the The Finnish Institute for Health and Welfare for the Health 2000 Survey were applied to the analyses to account for the sampling and non-participation of the survey participants. Appropriate statistical methods were applied to the analyses, see Methods: Statistical methods.

**25. Effect of engagement with patients and others affected by the study**

Report on any difference patient/service recipient, general public, community, or stakeholder involvement made to the approach or findings of the study.

Not Applicable.

**Discussion**

**26. Study findings, limitations, generalizability, and current knowledge**

Report key findings, limitations, ethical, or equity considerations not captured and how these could impact patients, policy, or practice.

See Methods: Ethics Approval and Informed Consent, and Discussion.

**Other Relevant Information**

**27. Source of funding**

Describe how the study was funded and any role of the funder in the identification, design, conduct, and reporting of the analysis.

This study was supported by Tampereen seudun Näkövammaisten tukisäätiö s.r, Tampere, Finland; Glaukooma Tukisäätiö Lux s.r, Helsinki, Finland; Elsemay Björn Fund, Helsinki, Finland; Finnish Federation of the Visually Impaired, Helsinki, Finland; Päivikki ja Sakari Sohlbergin Säätiö, Helsinki, Finland; and Juho Vainion Säätiö, Helsinki, Finland. The funders had no role in the design and conduct of the study; collection, management, analysis, and interpretation of the data; preparation, review, or approval of the manuscript; and decision to submit the manuscript for publication.

**28. Conflicts of interest**

Report authors’ conflicts of interest according to journal or International Committee of Medical Journal Editors requirements.

Authors declare no conflicts of interest.
