# Supplementary material for: Price tag of glaucoma care is minor compared with the total direct and indirect costs of glaucoma: Results from nationwide survey and register data
Source: PLoS One. 2023 Dec 20;18(12):e0295523. doi: 10.1371/journal.pone.0295523 (PMC10732367; doi:10.1371/journal.pone.0295523)
Supplement: S1 Table — (DOCX) [file pone.0295523.s002.docx]

**S1 Table.** **Direct and indirect costs in Finland in 2011 and 2019**

| **Health care resource** | **Cost per person (EUR)** | | **Reference** |
| --- | --- | --- | --- |
|  | **2011** | **2019^a^** |  |
| Secondary/tertiary hospital ward day | 737 | 905 | [20] |
| Secondary/tertiary hospital ophthalmic ward day | 873 | 1072 | [20] |
| Secondary/tertiary care ambulatory visit to doctor | 264 | 324 | [20] |
| Secondary/tertiary care ambulatory visit to eye clinic | 199 | 244 | [20] |
| Primary health care doctor visit (including collateral costs such as laboratory, imaging, and general costs) |  |  | [20] |
| during office hours | 110 | 135 |  |
| on emergency duty | 96 | 118 |  |
| Private practitioner visit (administrative payment added) | 66 | 81 | [20] |
| Occupational doctor visit | 77 | 95 | [20] |
| Occupational nurse visit | 28 | 34 | [20] |
| Home care nurse visit | 110 | 135 | [20] |
| Outpatient nurse visit | 48 | 59 | [20] |
| Travel cost per outpatient visit |  |  | [21] |
| Southern Finland | 18 | 22 |  |
| Western Finland | 22 | 27 |  |
| Central Finland | 24 | 29 |  |
| Eastern Finland | 30 | 37 |  |
| Northern Finland | 41 | 50 |  |
| Annual pension | 16,428 | 20,178 | Finnish Centre for Pensions |
| Annual gross domestic product | 40,078 | 49,226 | Statistics Finland |

^a^Converted from year 2011.
