# Supplementary material for: Price tag of glaucoma care is minor compared with the total direct and indirect costs of glaucoma: Results from nationwide survey and register data
Source: PLoS One. 2023 Dec 20;18(12):e0295523. doi: 10.1371/journal.pone.0295523 (PMC10732367; doi:10.1371/journal.pone.0295523)
Supplement: S2 Table — (DOCX) [file pone.0295523.s003.docx]

**Table S2. Mean annual direct health care costs with 95% confidence intervals (CIs) in the Finnish population aged 30 years and older at the 2019 cost level**

|  | **Annual costs per person (EUR)** | | | | | | |
| --- | --- | --- | --- | --- | --- | --- | --- |
|  | **Hospitalizations** | | **Outpatient visits** | | **Outpatient health care services** | **Outpatient travels** | |
|  | **Eye (95% CI)** | **Non-eye (95% CI)** | **Eye (95% CI)** | **Non-eye (95% CI)** | **All (95% CI)** | **Eye (95% CI)** | **Non-eye (95% CI)** |
| ***Non-adjusted costs*** |  |  |  |  |  |  |  |
| Glaucoma negatives | 22 (17–26) | 4,001 (3,488–4,514) | 16 (14–17) | 376 (351–400) | 434 (402–467) | 2 (2–2) | 36 (33–39) |
| Glaucoma, all | 175 (119–230) | 14,915 (9,844–19,987) | 162 (124–201) | 511 (398–623) | 722 (572–871) | 20 (16–25) | 46 (37–55) |
| Glaucoma, medication | 207 (134–280) | 12,436 (7,857–17,016) | 186 (135–237) | 508 (411–605) | 729 (561–898) | 24 (17–30) | 47 (37–56) |
| Glaucoma, operated | 226 (96–357) | 17,866 (6,858–28,874) | 215 (141–290) | 672 (365–978) | 846 (484–1,209) | 29 (19–39) | 57 (34–79) |
| ***Adjusted for age and sex*** |  |  |  |  |  |  |  |
| Glaucoma negatives | 24 (23–24) | 4,415 (4,312–4,519) | 16 (16–17) | 379 (370–388) | 451 (441–462) | 2 (2–2) | 36 (35–37) |
| Glaucoma, all | 152 (130–173) | 6,141 (5,274–7,007) | 209 (180–239) | 610 (524–696) | 798 (685–910) | 24 (21–27) | 50 (43–57) |
| Glaucoma, medication | 178 (149–208) | 5,601 (4,677–6,526) | 209 (174–243) | 400 (334–466) | 644 (537–750) | 25 (21–29) | 35 (29–40) |
| Glaucoma, operated | 154 (114–193) | 6,712 (4,999–8,424) | 177 (132–222) | 1,074 (800–1,348) | 1,085 (808–1,362) | 22 (16–27) | 79 (59–99) |

All eye- and non-eye-related adjusted and non-adjusted direct annual costs per person were significantly higher in the three glaucoma groups compared with glaucoma negatives (p < 0.001), but there were no significant differences within the three glaucoma groups.
