# Supplementary material for: Price tag of glaucoma care is minor compared with the total direct and indirect costs of glaucoma: Results from nationwide survey and register data
Source: PLoS One. 2023 Dec 20;18(12):e0295523. doi: 10.1371/journal.pone.0295523 (PMC10732367; doi:10.1371/journal.pone.0295523)
Supplement: S3 Table — (DOCX) [file pone.0295523.s004.docx]

**S3 Table. Mean non-adjusted direct health care costs in glaucoma patients with different treatments at the 2019 cost level**

|  | **Annual costs per person (EUR)** | | | | |
| --- | --- | --- | --- | --- | --- |
|  | **Hospitalizations** | | **Outpatient visits** | | **Outpatient health care services** |
|  | Eye | Non-eye | Eye | Non-eye | All |
| Only medication | 140 | 9,496 | 125 | 404 | 557 |
| Only operated | 62 | 20,488 | 77 | 776 | 689 |
| Both medication and operated | 242 | 11,760 | 222 | 440 | 689 |
| No medication or operation | 70 | 16,411 | 75 | 210 | 498 |

Hospitalization and outpatient visit data were collected during 1999–2011 and outpatient health care services in 2000.
