# Supplementary material for: Price tag of glaucoma care is minor compared with the total direct and indirect costs of glaucoma: Results from nationwide survey and register data
Source: PLoS One. 2023 Dec 20;18(12):e0295523. doi: 10.1371/journal.pone.0295523 (PMC10732367; doi:10.1371/journal.pone.0295523)
Supplement: S4 Table — (DOCX) [file pone.0295523.s005.docx]

**Table S4. Mean indirect costs** **with 95% confidence intervals (CIs) in the Finnish population aged 30–64 years at the 2019 cost level**

|  | **Costs per person retired prematurely (EUR)** | |
| --- | --- | --- |
|  | **Premature retirement (95% CI)** | **Productivity loss (95% CI)** |
| Glaucoma negatives | 154,185 (147,882–160,487) | 376,151 (360,776–391,527) |
| Glaucoma, all | 194,823 (136,258–253,389) | 475,294 (332,417–618,172) |
| Glaucoma, medication | 184,947 (122,472–247,421) | 451,198 (298,784–603,613) |
| Glaucoma, operated | 276,467 (120,735–432,199) | 674,473 (294,547–1,054,399) |

No statistical differences were observed in personal indirect costs between the three glaucoma groups and glaucoma negatives and within the three glaucoma groups. Data were collected during 1999–2011.
