# Supplementary material for: Price tag of glaucoma care is minor compared with the total direct and indirect costs of glaucoma: Results from nationwide survey and register data
Source: PLoS One. 2023 Dec 20;18(12):e0295523. doi: 10.1371/journal.pone.0295523 (PMC10732367; doi:10.1371/journal.pone.0295523)
Supplement: S5 Table — (DOCX) [file pone.0295523.s006.docx]

**S5 Table. Multivariable regression analysis examining the impact of glaucoma, age, sex, and non-eye-related co-morbidities on total annual direct health care costs in population aged 30 years and older at the 2019 cost level**

|  | **B coefficient** | **Marginal mean (EUR)** | **Marginal mean contrast (EUR)** | **P value** |  |  | **B coefficient** | **Marginal mean (EUR)** | **Marginal mean contrast (EUR)** | **P value** |  |  | **B coefficient** | **Marginal mean (EUR)** | **Marginal mean contrast (EUR)** | **P value** |
| --- | --- | --- | --- | --- | --- | --- | --- | --- | --- | --- | --- | --- | --- | --- | --- | --- |
| Constant | 4.700 |  |  | < 0.001 |  | Constant | 4.705 |  |  | < 0.001 |  | Constant | 4.704 |  |  | < 0.001 |
| Age | 0.052 |  |  | < 0.001 |  | Age | 0.052 |  |  | < 0.001 |  | Age | 0.052 |  |  | < 0.001 |
| Male sex | –0.170 | 40,588 | –7,523 | 0.044 |  | Male sex | –0.172 | 39,286 | –7,351 | 0.044 |  | Male sex | –0.174 | 43,554 | –8,265 | 0.042 |
| Glaucoma, all | 0.112 | 46,746 | 4,973 | 0.31 |  | Glaucoma, medication | 0.036 | 43,591 | 1,561 | 0.79 |  | Glaucoma, operated | 0.283 | 54,721 | 13,478 | 0.13 |
| Heart disease | 0.256 | 50,226 | 11,347 | 0.011 |  | Heart disease | 0.257 | 48,666 | 11,019 | 0.009 |  | Heart disease | 0.257 | 54,028 | 12,257 | 0.012 |
| Pulmonary disease | 0.104 | 46,538 | 4,579 | 0.22 |  | Pulmonary disease | 0.112 | 45,260 | 4,780 | 0.19 |  | Pulmonary disease | 0.107 | 50,120 | 5,091 | 0.22 |
| Vascular disease | –0.022 | 43,712 | –960 | 0.80 |  | Vascular disease | –0.018 | 42,413 | –785 | 0.83 |  | Vascular disease | –0.026 | 46,891 | –1,238 | 0.77 |
| Musculoskeletal condition | 0.224 | 49,431 | 9,928 | 0.001 |  | Musculoskeletal condition | 0.221 | 47,804 | 9,477 | 0.002 |  | Musculoskeletal condition | 0.225 | 53,165 | 10,715 | 0.002 |
| Hypertension | 0.050 | 45,316 | 2,226 | 0.51 |  | Hypertension | 0.049 | 43,874 | 2,115 | 0.53 |  | Hypertension | 0.049 | 48,696 | 2,350 | 0.53 |
| Diabetes | 0.650 | 61,173 | 29,252 | < 0.001 |  | Diabetes | 0.645 | 59,079 | 28,068 | < 0.001 |  | Diabetes | 0.633 | 65,184 | 30,560 | < 0.001 |
| Psychiatric disorder | 1.175 | 79,505 | 54,943 | < 0.001 |  | Psychiatric disorder | 1.174 | 76,988 | 53,191 | < 0.001 |  | Psychiatric disorder | 1.167 | 85,142 | 58,636 | < 0.001 |
| Parkinson's disease | 1.359 | 87,178 | 64,778 | < 0.001 |  | Parkinson's disease | 1.362 | 84,557 | 62,889 | < 0.001 |  | Parkinson's disease | 1.358 | 93,675 | 69,583 | < 0.001 |
| Cancer | 0.377 | 53,348 | 16,745 | < 0.001 |  | Cancer | 0.390 | 52,028 | 16,812 | < 0.001 |  | Cancer | 0.374 | 57,282 | 17,883 | < 0.001 |

Tweedie distribution using gamma with log link scale response was applied to the model. The analysis was based on participants with information available for all predictors (*n* = 6728–6861). The age was standardized for the average age of glaucomatous population in Finland (71.9 years) for the marginal means and contrasts. Marginal mean contrasts equal the difference between those with a medical condition (or of male sex) and those without a medical condition (or of female sex) standardized for all other factors. Statistical significance was calculated for both the B coefficients and marginal mean contrasts.
