# Supplementary material for: Correction: Price tag of glaucoma care is minor compared with the total direct and indirect costs of glaucoma: Results from nationwide survey and register data
Source: PLoS One. 2025 Jan 30;20(1):e0318723. doi: 10.1371/journal.pone.0318723 (PMC11781623; doi:10.1371/journal.pone.0318723)
Supplement: S1 Table — (DOCX) [file pone.0318723.s001.docx]

**S1 Table.** **Direct and indirect costs in Finland in 2011 and 2019**

| **Health care resource** | **Cost per person (EUR)** | | **Reference** |
| --- | --- | --- | --- |
|  | **2011** | **2019^a^** |  |
| Secondary/tertiary hospital ward day | 737 | 905 | [21] |
| Secondary/tertiary hospital ophthalmic ward day | 873 | 1072 | [21] |
| Secondary/tertiary care ambulatory visit to doctor | 264 | 324 | [21] |
| Secondary/tertiary care ambulatory visit to eye clinic | 199 | 244 | [21] |
| Primary health care doctor visit (including collateral costs such as laboratory, imaging, and general costs) |  |  | [21] |
| during office hours | 110 | 135 |  |
| on emergency duty | 96 | 118 |  |
| Private practitioner visit (administrative payment added) | 66 | 81 | [21] |
| Occupational doctor visit | 77 | 95 | [21] |
| Occupational nurse visit | 28 | 34 | [21] |
| Home care nurse visit | 110 | 135 | [21] |
| Outpatient nurse visit | 48 | 59 | [21] |
| Annual pension | 16,428 | 20,178 | Finnish Centre for Pensions |
| Annual gross domestic product | 36,746 | 45,133 | Statistics Finland |
| Travel cost per outpatient visit | **2017** | **2019^b^** | [22] |
| Southern Finland | 18 | 19 |  |
| Western Finland | 22 | 23 |  |
| Central Finland | 24 | 25 |  |
| Eastern Finland | 30 | 32 |  |
| Northern Finland | 41 | 44 |  |

^a^Converted from year 2011.

^b^Converted from year 2017.
