# Supplementary material for: Correction: Price tag of glaucoma care is minor compared with the total direct and indirect costs of glaucoma: Results from nationwide survey and register data
Source: PLoS One. 2025 Jan 30;20(1):e0318723. doi: 10.1371/journal.pone.0318723 (PMC11781623; doi:10.1371/journal.pone.0318723)
Supplement: S4 Table — (DOCX) [file pone.0318723.s003.docx]

**Table S4. Mean indirect costs** **with 95% confidence intervals (CIs) in the Finnish population aged 30–64 years at the 2019 cost level**

|  | **Costs per person retired prematurely (EUR)** | |
| --- | --- | --- |
|  | **Premature retirement (95% CI)** | **Productivity loss (95% CI)** |
| Glaucoma negatives | 154,185 (147,882–160,487) | 344,879 (330,782–358,976) |
| Glaucoma, all | 194,823 (136,258–253,389) | 435,779 (304,780–566,779) |
| Glaucoma, medication | 184,947 (122,472–247,421) | 413,687 (273,944–553,430) |
| Glaucoma, operated | 276,467 (120,735–432,199) | 618,399 (270,059–966,739) |

No statistical differences were observed in personal indirect costs between the three glaucoma groups and glaucoma negatives and within the three glaucoma groups. Data were collected during 1999–2011.
